# Molecular Neuropathology of Gliomas

**DOI:** 10.3390/ijms10010184

**Published:** 2009-01-07

**Authors:** Markus J. Riemenschneider, Guido Reifenberger

**Affiliations:** Department of Neuropathology, Heinrich-Heine-University, Moorenstr. 5, 40225 Duesseldorf, Germany

**Keywords:** Glioblastoma, oligodendroglioma, ependymoma, molecular diagnostics, genetics, MGMT, 1p, 19q, biomarker, profiling

## Abstract

Gliomas are the most common primary human brain tumors. They comprise a heterogeneous group of benign and malignant neoplasms that are histologically classified according to the World Health Organization (WHO) classification of tumors of the nervous system. Over the past 20 years the cytogenetic and molecular genetic alterations associated with glioma formation and progression have been intensely studied and genetic profiles as additional aids to the definition of brain tumors have been incorporated in the WHO classification. In fact, first steps have been undertaken in supplementing classical histopathological diagnosis by the use of molecular tests, such as *MGMT* promoter hypermethylation in glioblastomas or detection of losses of chromosome arms 1p and 19q in oligodendroglial tumors. The tremendous progress that has been made in the use of array-based profiling techniques will likely contribute to a further molecular refinement of glioma classification and lead to the identification of glioma core pathways that can be specifically targeted by more individualized glioma therapies.

## 1. Introduction

Primary tumors of the central nervous system (CNS) account for approximately 2–3% of all cancers. In Western countries, the annual incidence is approximately 15 patients per 100,000 population and the prevalence has been estimated to approximately 69 patients per 100,000 population [1]. In children, CNS tumors are the second most common form of cancer after leukemia. Primary CNS tumors comprise a heterogeneous group of benign and malignant neoplasms, the most common of which are tumors of glial cells, collectively referred to as gliomas [1]. Gliomas are histologically classified according to the World Health Organization (WHO) classification of tumors of the nervous system [2], which combines tumor typing with the assignment of a defined malignancy grade (Table 1). Four histological malignancy grades have been defined that reach from benign tumors of WHO grade I to highly malignant tumors of WHO grade IV. Pilocytic astrocytoma, the most common glioma in childhood, is the prototype of a WHO grade I lesion. On the other end of the spectrum, WHO grade IV is assigned to glioblastoma, the most common and most malignant type of glioma, which preferentially manifests in adults with a peak incidence between 50 and 60 years (Table 1).

The histological classification and grading to date still represents the most reliable and meaningful indicator for the biological and clinical behavior of gliomas as well as patient outcome. Patients with WHO grade I tumors can usually be cured by surgical resection. WHO grade II tumors -though still exhibiting a rather slow growth- nearly invariably recur after resection and bear the inevitable tendency to progress to anaplastic gliomas of WHO grade III or secondary glioblastomas of WHO grade IV. Thus, median survival of patients with WHO grade II gliomas is in the range of only 5 – 8 years after diagnosis. Anaplastic gliomas (WHO grade III) are rapidly growing malignant tumors that, in addition to surgery, require aggressive adjuvant treatment with radio- and/or chemotherapy. Median survival time is just 2–3 years after diagnosis, except for the subgroup of patients with anaplastic oligodendroglial tumors, who often do better, in particular when their tumors carry a prognostically favorable combined deletion of chromosomal arms 1p and 19q, as outlined below [3, 4]. Glioblastoma patients exhibit a rapid disease progression despite multimodal aggressive treatment and median survival time -with few exceptions- is in the range of just one year after initial diagnosis.

Research efforts of the past decades identified a plethora of cytogenetic and molecular genetic alterations in gliomas that may be exploited to facilitate glioma classification, especially in cases that exhibit inconclusive or borderline histological features. In this review, we will provide a comprehensive overview of the cytogenetic and molecular alterations that are associated with the individual glioma entities and describe their role in regard to glioma initiation and progression. Special emphasis will be put on the introduction of those molecular biomarkers that have been established in glioma diagnostics, namely *MGMT* promoter hypermethylation in glioblastomas and deletion of chromosome arms 1p and 19q in patients with oligodendroglial tumors. Finally, we will highlight the tremendous impact that high-troughput profiling techniques, such as gene expression profiling, array-CGH, methylation profiling and large-scale sequencing, have on the genome-wide identification of novel tumor suppressor and proto-oncogenes. Integrative analysis over the different platforms will not only expand our knowledge of the molecular basis of gliomas but also pave the way to a molecularly refined glioma classification and facilitate the detection of “genes in context”, i.e. pathways of gliomagenesis that can be specifically targeted with individualized therapies.

## 2. Cytogenetic and molecular changes within individual glioma types

### 2.1. Diffusely infiltrating astrocytic gliomas

#### 2.1.1. Diffuse astrocytoma (WHO grade II)

The most common chromosomal abnormality in diffuse astrocytomas of WHO grade II is trisomy 7 or at least a gain of 7q, which has been detected by comparative genomic hybridization in 50% of the cases [5, 6]. However, the relevant target genes on chromosome 7 have not yet been identified. Further chromosomal aberrations comprise losses on 22q, 19q, 13q, 10p, 6 and the sex chromosomes as well as gains on 5p, 9 and 19p [7].

**Figure 1.**
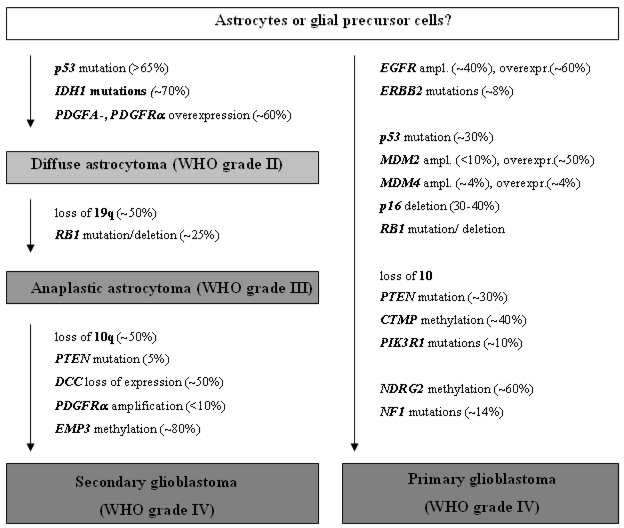
Schematic representation of the molecular pathogenesis and progression of diffusely infiltrating astrocytic gliomas.

The most common molecular alterations are mutations of the TP53 tumor suppressor gene at 17q13.1 in about 60% of cases as well as the just recently identified codon 132 mutations of the isocitrate dehydrogenase 1 (IDH1) gene in about 70% of diffuse astrocytomas [8–10] (Figure 1).

Even higher frequencies of *TP53* mutations of up to 80% are detectable in the gemistocytic astrocytoma variant [11]. *TP53* mutations are regarded as one of the earliest events in the tumorigenesis of diffuse astrocytomas, since in most cases they are already present in the first biopsy and their frequency does not increase in recurrences. *TP53* mutations in diffuse astrocytomas are commonly associated with loss of heterozygosity (LOH) at polymorphic loci on 17p resulting in complete loss of wild-type p53 in the tumor cells.

In tumors without *TP53* alterations, the *p14^ARF^* gene at 9p21, the gene product of which regulates MDM2-mediated degradation of p53, is frequently methylated and transcriptionally downregulated [12]. Other genes that are frequently epigenetically silenced in diffuse astrocytomas include the *MGMT* gene at 10q26 [12], the protocadherin-gamma subfamily A11 (*PCDH-gamma-A11*) gene at 5q31 [13], and the *EMP3* gene at 19q13 [14]. Interestingly, *MGMT* hypermethylation was found to be associated with *TP53* mutation but is mutually exclusive to *p14^ARF^* hypermethylation [12].

Another common alteration in diffuse astrocytomas is overexpression of the platelet-derived growth factor receptor alpha (PDGFRA) and its ligand PDGFalpha [15]; *PDGFRA* amplification, however, is restricted to a small subset of high-grade gliomas, in particular glioblastomas [16] (Figure 1).

#### 2.1.2. Anaplastic astrocytoma (WHO grade III)

Anaplastic astrocytomas show gains of chromosome 7 and *TP53* mutations as well as *IDH1* mutations at a similar frequency as diffuse astrocytomas. In addition, these tumors bear frequent allelic losses on chromosomes 6, 9p, 11p, 19q and 22q. The *CDKN2A, p14^ARF^* and *CDKN2B* tumor suppressor genes are important targets for genetic and or epigenetic inactivation, with inactivation of *p14^ARF^* serving as an alternative means to impair the p53 pathway in cases without *TP53* mutations [8]. The gene products of *CDKN2A,* i.e. p16^INK4a^, and *CDKN2B*, i.e. p15^INK4b^, function as negative regulators of the cell cycle at the G1/S-phase transition by inhibiting the formation of complexes between D-type cyclins and the cyclin dependent kinases Cdk4 or Cdk6. The *CDK4* gene at 12q13–q14 is amplified and overexpressed in about 10% of anaplastic astrocytomas [17], preferentially in tumors without *CDKN2A* deletion or mutation [18, 19].

In addition, about 25% of anaplastic astrocytomas carry mutations in the retinoblastoma gene (*RB1*) [18] (Figure 1). In contrast to glioblastomas, allelic losses on 10q and mutation of the *PTEN* tumor suppressor gene on 10q23 are rare in anaplastic astrocytomas (<10% of cases). However, when present, *PTEN* mutation indicates a poor prognosis [20].

#### 2.1.3. Glioblastoma (WHO grade IV)

Glioblastomas usually carry multiple chromosomal and genetic aberrations. In addition to the above listed aberrations found in diffuse and anaplastic astrocytomas, glioblastomas typically exhibit loss of genetic material from chromosome 10. Interestingly, molecular genetic analyses over the past years have shed light on two different genetic pathways in glioblastoma formation establishing the concept of primary and secondary glioblastomas [21] (Figure 1). Primary glioblastomas arise *de novo* without the clinical history of a lower grade precursor lesion. Primary glioblastomas exhibit frequent *EGFR* amplification, homozygous deletion of *CDKN2A* and *p14^ARF^*, *CDK4* amplification, *MDM2* or *MDM4* amplification, *RB1* mutation/homozygous deletion, monosomy 10 and *PTEN* mutation [21–25]. *EGFR* amplification is particularly frequent in primary glioblastomas with a small cell, highly anaplastic histological phenotype [26]. *TP53* mutation is found in less than 30% of primary glioblastomas. In contrast, secondary glioblastomas arise from a lower-grade precursor lesion and carry *TP53* and *IDH1* mutations in more than two thirds of the cases [10, 21]. Also, allelic losses on 19q and 13q, promoter hypermethylation of the *RB1* gene, and overexpression of *PDGFRA* are more common in secondary glioblastomas [21, 22, 27]. Amplification of *EGFR* or *MDM2*, *PTEN* mutation as well as homozygous *CDKN2A* or *p14^ARF^* deletions are all rare in secondary glioblastomas. Also epigenetic silencing of various genes has been described as overrepresented in either primary (*NDRG2*) or secondary (*MGMT* and *EMP3*) glioblastomas [21, 28, 29]. Taken together, these data clearly indicate that primary and secondary glioblastomas represent genetically different disease entities (Figure 1). Nevertheless, both entities share comparable histological features and an equally poor prognosis. The fact that the different molecular alterations converge on the same downstream cellular pathways, namely the p53, pRb1, PTEN/PI3K/AKT and mitogen-activated protein kinase pathways, and thereby lead to similar functional consequences, may explain this phenomenon [7, 30, 31].

**Breakout box 1: *MGMT* hypermethylation as a predictive marker in glioblastomas.**

The *MGMT* (O^6^-methylguanine–DNA methyltransferase) gene on chromosome band 10q26 encodes a DNA repair protein that removes alkyl groups from the O^6^ position of guanine, an important site of DNA alkylation [32]. Chemotherapy-induced alkylation in this location triggers cytotoxicity and apoptosis. High levels of the MGMT repair protein thus may counteract the therapeutic effect of alkylating agents and thereby lead to treatment failure. Epigenetic silencing of *MGMT* by means of promoter hypermethylation is present in about 40% of primary glioblastomas and has been identified as the main mechanism reducing MGMT expression and thereby diminishing its DNA repair activity. Importantly, *MGMT* promoter methylation has been associated with response of glioblastomas to alkylating chemotherapy using nitrosourea compounds [33], temozolomide [34], or a combination of both [35]. Based on *MGMT* promoter methylation analysis in glioblastomas from patients treated in a large prospective clinical trial, patients whose tumors had a methylated *MGMT* promoter survived significantly longer as compared to patients whose tumors lacked *MGMT* promoter methylation when treated with combined radio-/ chemotherapy [34]. In glioblastoma patients treated with radiotherapy alone, *MGMT* promoter methylation did not significantly influence survival, thus indicating that the *MGMT* promoter status is a predictive factor for response to chemotherapy. As *MGMT* promoter methylation can be tested by methylation-specific polymerase chain reaction (MSP) analysis or other methods, *MGMT* testing is now increasingly being requested not only for patients in clinical trials but also in routine diagnostics.

Giant cell glioblastomas carry *TP53* mutations in up to 90% and *PTEN* mutations in 30–40% of cases, thus combining molecular features of both primary and secondary glioblastoma. *EGFR* amplification and homozygous deletions of *CDKN2A* and *p14^ARF^* are usually absent [36, 37].

The molecular genetics of gliosarcoma is fairly similar to that of primary glioblastoma, except for *EGFR* amplification, which seems to be less frequent [38]. Microdissection of the gliomatous and sarcomatous tumor components followed by CGH analysis revealed common genetic aberrations in both components, thus arguing for a monoclonal origin of both histological aspects of gliosarcomas [39].

Rare glioblastomas may exhibit metaplastic histologic features with adenoid or squamous cell epithelial differentiation, bone or cartilage formation as well as xanthomatous or adipocytic changes. However, none of these alterations are as yet known to be reflected by distinct alterations on the molecular level [40–44].

### 2.2. Astrocytic gliomas with more circumscribed growth

#### 2.2.1. Pilocytic astrocytoma (WHO grade I)

**Figure 2.**
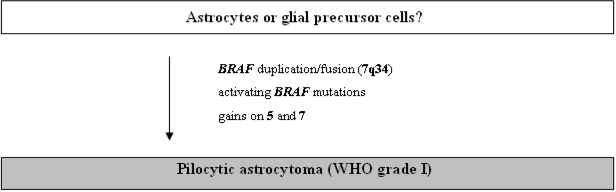
Schematic representation of the molecular pathogenesis of pilocytic astrocytomas.

In pilocytic astrocytomas molecular and cytogenetic investigations have identified far less chromosomal and genetic alterations than in the diffusely infiltrating astrocytomas described above. Chromosomal comparative genomic hybridization (CGH) studies in 48 pilocytic astrocytomas revealed chromosomal imbalances only in a small subgroup of seven neoplasms [45] with gain of 9q34.1-qter constituting the most common abnormality. Recurrent trisomies of chromosomes 5 and chromosome 7 have been reported in another study employing array-CGH on 53 pilocytic astrocytomas [46] (Figure 2).

In patients with neurofibromatosis type 1 (NF1) pilocytic astrocytomas are the most common gliomas. In this setting, pilocytic astrocytomas are typically located in the optic nerve, often bilaterally, and carry allelic losses at the *NF1* tumor suppressor gene locus at 17q11.2 [47]. Sporadic pilocytic astrocytomas, in contrast, rarely demonstrate allelic loss at the *NF1* locus [47]. Furthermore, neither *NF1* mutations nor loss of *NF1* mRNA expression were found in sporadic pilocytic astrocytomas [48]. Losses on 17p and mutations in the *TP53* tumor suppressor genes that are observed in diffuse astrocytomas are not a common feature in pilocytic astrocytomas [47, 49, 50]. In contrast, circumscribed duplication of the *BRAF* gene at 7q34 resulting in increased BRAF expression has been identified as a common aberration [46, 51–53]. *BRAF* duplication results in an in-frame fusion gene incorporating the kinase domain of the *BRAF* oncogene with constitutive BRAF kinase activity. A small subset of tumors alternatively carries activating *BRAF* mutations, thus implicating this gene as an important proto-oncogene in these tumors (Figure 2).

Recently, the pilomyxoid astrocytoma has been recognized as a histologically and clinically distinct variant of pilocytic astrocytoma with a less favorable prognosis [54]. Local recurrences as well as cerebrospinal spread occur more often in pilomyxoid tumors than in pilocytic astrocytomas. The WHO classification thus assigns these tumors to WHO grade II. Data on whether the histological and prognostic differences between pilocytic and pilomyxoid astrocytomas are also reflected by different genetic alterations at the molecular level are still scarce. A recent study employing gene expression profiling of NF1-associated and sporadic pilocytic and pilomyxoid astrocytomas identified aldehyde dehydrogenase 1 family member L1 (*ALDH1L1*) as an underexpressed candidate biomarker in more aggressive tumor subtypes [55].

#### 2.2.2. Pleomorphic xanthoastrocytoma (WHO grade II)

Loss on chromosome 9 is the most common genomic imbalance in pleomorphic xanthoastrocytoma (PXA), which is detectable by CGH analysis in 50% of cases. Other losses affect chromosomes 17 (10%), 8, 18 and 22 (4% each). Chromosomal gains could be identified on chromosomes X (16%), 7, 9q, 20 (8% each), 4, 5 and 19 (4% each) [56].

*TP53* mutations are seen in a small fraction of tumors (<10% of cases), while 1p/19q losses as well as amplification of *EGFR*, *CDK4* and *MDM2* are absent [57, 58]. In contrast, homozygous deletion of the tumor suppressor genes *CDKN2A*, *p14^ARF^* and *CDKN2B* on 9p21.3 is common in PXA [56]. Interestingly, transcript levels of the *TSC1* gene on 9q were found to be consistently low in PXA; however, the causative mechanism still remains unclear, as there was no evidence for *TSC1* mutations or promoter methylation [56].

#### 2.2.3. Subependymal giant cell astrocytoma (WHO grade I)

Biallelic inactivation of either the *TSC1* or the *TSC2* tumor suppressor gene is a hallmark for these tumors [59]. Since the corresponding gene products have an inhibitory function on the mTOR pathway, their mutational inactivation leads to aberrant activation of mTOR signaling, which in turn represents an interesting novel target for specific pharmacologic inhibition. A comparative genomic hybridization study on subependymal giant cell astrocytomas indicated that chromosomal imbalances are either rare or completely absent [40].

### 2.3. Oligodendrogliomas and mixed gliomas

#### 2.3.1. Oligodendroglioma (WHO grade II)

**Figure 3.**
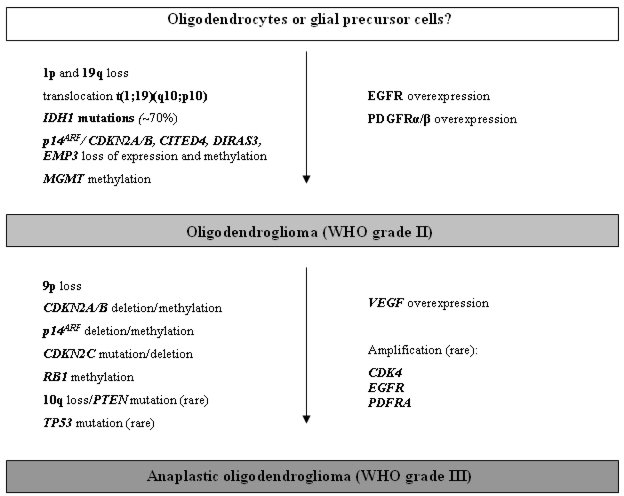
Schematic representation of the molecular pathogenesis and progression of oligodendrogliomas.

Despite extensive research efforts, oligodendroglioma-specific immunohistochemical markers could not yet be identified [60–62]. However, the most characteristic genetic feature of oligodendroglial tumors is the combined loss of alleles on 1p and 19q, which may be detected in up to two thirds of the cases [63] (Figure 3).

Two independent studies reported that an unbalanced t(1;19)(q10;p10) translocation, with the chromosomal breakpoints located close to the centromers of both chromosomes, serves as the cytogenetic mechanism responsible for the frequent co-deletions of both chromosome arms in oligodendrogliomas [64, 65]. The oligodendroglioma-associated tumor suppressor genes on 1p and 19q, however, have not yet been identified. Recent studies indicate that more than one gene from 1p may be aberrant in oligodendrogliomas. The *NOTCH2* gene maps closest to the breakpoint region on 1p13–p11 and may function as a putative oligodendroglioma-associated tumor suppressor gene considering that intragenic homozygous deletions have been found in two tumors [66]. But also a number of other candidate tumor suppressor genes from different regions on 1p have been proposed, including *TP73* (1p36.3), the calmodulin-binding transcription activator 1 gene (*CAMTA1*, 1p36), the DNA fragmentation factor subunit ß gene (*DFFB*, 1p36), *SHREW1* (1p36.32), *CITED4* (1p34.2), *RAD54* (1p32), *CDKN2C* (1p32), and *DIRAS3* (1p31) [67–73]. Candidate tumor suppressor genes on 19q include the *p190RhoGAP* gene at 19q13.3 [74], the myelin-related epithelial membrane protein gene 3 (*EMP3*) at 19q13.3 [75], *ZNF342*, a zinc-finger transcription factor gene at 19q13 [76], and the maternally imprinted *PEG3* gene at 19q13.4 [77].

In addition to 1p/19q deletions, several other genetic and epigenetic alterations have been described in oligodendrogliomas (Figure 3). Less frequent cytogenetic alterations are gains on chromosomes 7 and 19p as well as losses on chromosomes 4, 6, 9p, 10q, 11p, 14, 18q, and 22q [71, 78]. Some of these chromosomal imbalances have been suggested as being linked to poor outcome, including gain of 7p and 8q as well as losses on 9p and 18q [79]. Both oligodendrogliomas of WHO grade II as well as anaplastic oligodendrogliomas (WHO grade III) share the high frequency of *IDH1* mutations (~70%) with astrocytic gliomas [9]. Allelic losses on 17p and *TP53* gene mutations are rare in low-grade oligodendrogliomas as they are mutually exclusive to the frequent 1p/19q losses. Nevertheless functional inactivation of the p53 pathway in oligodendrogliomas may be achieved by means of epigenetic silencing of the *p14^ARF^* gene, which thereby loses its ability to bind Mdm2 and to prevent the Mdm2-mediated degradation of p53 [80, 81].

**Breakout box 2: Combined deletion of chromosome arms 1p and 19q as a biomarker in (anaplastic) oligodendrogliomas.**

Anaplastic oligodendrogliomas with loss on 1p, or combined losses on 1p and 19q, usually respond favorably to chemotherapy, with about half of such tumors showing complete neuroradiological responses [91]. On the contrary, only 25% of the anaplastic oligodendrogliomas that lack these genetic changes respond to PCV. Furthermore, patients whose tumors have 1p or combined 1p and 19q losses show significantly longer survival (mean survival of > 10 years) as compared to patients whose tumors lack these genetic changes (mean survival of approximately 2 years) [91, 92]. Initially reported by Cairncross and colleagues in 1998 [91], several retrospective studies have confirmed combined deletions of 1p and 19q as an independent marker of favorable response to radio- and chemotherapy as well as prolonged survival in patients with high-grade oligodendroglial tumors [87, 88, 92, 93]. Also, two prospective and randomized phase III trials involving 368 patients and 289 patients, respectively, confirmed the prognostic value of 1p/19q deletion in patients with anaplastic oligodendroglial tumors [3, 4]. Owing to the major prognostic significance of the 1p/19q status in patients with anaplastic gliomas treated with radio- and/or chemotherapy, ongoing prospective trials are no longer stratifying anaplastic glioma patients according to histological type but according to the 1p/19q deletion status. Thus, it is likely that molecular testing for 1p/19q deletion will become a routine adjunct to histology in the diagnostic assessment of anaplastic gliomas. The prognostic role of 1p/19q deletion in low-grade oligodendroglioma patients is less clear. Several recent studies independently reported that 1p deletion or 1p/19q co-deletion were also associated with a trend towards longer survival in low-grade oligodendroglioma patients [89, 92–95], and that in patients treated with temozolomide 1p loss was associated with objective treatment response [96, 97]. Nevertheless, contrary findings also have been reported, which could not corroborate combined 1p/19q loss as a sensitive prognostic biomarker in patients with oligodendroglial tumors who did not receive radiotherapy or chemotherapy [98].

Other genes hypermethylated in oligodendrogliomas include the tumor suppressors *CDKN2A*, *CDKN2B* and *RB1*, as well as *DAPK1* (death-associated protein kinase 1), *ESR1* (estrogen receptor 1), *THBS* (thrombospondin 1) and *TIMP3* (tissue inhibitor of metalloproteinase 3) [81–83]. Frequent promoter hypermethylation and reduced expression of the *MGMT* gene in oligodendrogliomas and consecutive impairment of MGMT-mediated DNA repair might in part contribute to the chemosensitivity of these neoplasms [84, 85].

Finally, oligodendrogliomas frequently demonstrate increased expression of growth factor receptors, such as EGFR and PDGFR [71, 86]. Platelet-derived growth factors A and B, as well as the corresponding receptors (PDGFR-alpha and PDGFR-beta) have been reported as constantly co-expressed in oligodendrogliomas suggesting an auto- and/or paracrine growth stimulatory activity of this signaling pathway [86] (Figure 3).

#### 2.3.2. Anaplastic oligodendroglioma (WHO grade III)

Malignant progression of oligodendroglial tumors is associated with the accumulation of multiple genetic abnormalities. Anaplastic oligodendrogliomas share the frequent loss of alleles on 1p and 19q with low-grade oligodendrogliomas, but additionally show frequent deletions on 9p and/or on chromosome 10 [87–89] (Figure 3). On 9p21, the tumor suppressor genes *CDKN2A*, *p14^AR^*^F^ and *CDKN2B* are homozygously deleted in up to one third of anaplastic oligodendrogliomas [71, 78]. Such deletions are particularly common in anaplastic oligodendrogliomas without 1p and 19q losses, but may also be present in 1p/19q-deleted cases. *PTEN* mutations occur in only about half of the cases with 10q loss, suggesting that there might be another progression-related target gene in this region [88, 89]. *PTEN* mutations and 10q deletions are more common in anaplastic oligodendrogliomas without 1p and 19q losses. Rare anaplastic oligodendrogliomas carry activating mutations in the *PIK3CA* gene [90].

Comparative genomic hybridization studies have uncovered that chromosomal imbalances on several other chromosomes occur at more than random frequency in anaplastic oligodendrogliomas, most notably losses on chromosomes 4, 6, 11, 13q, 18 and 22q as well as gains on chromosomes 7, 15 and 20 [71, 78]. Gene amplifications affecting the *EGFR*, *PDGFRA*, *CDK4*, *MDM4*, *MYC* or *MYCN* are uncommon (generally <10%) [71, 78].

#### 2.3.3. Oligoastrocytomas (WHO grade II or III)

**Figure 4.**
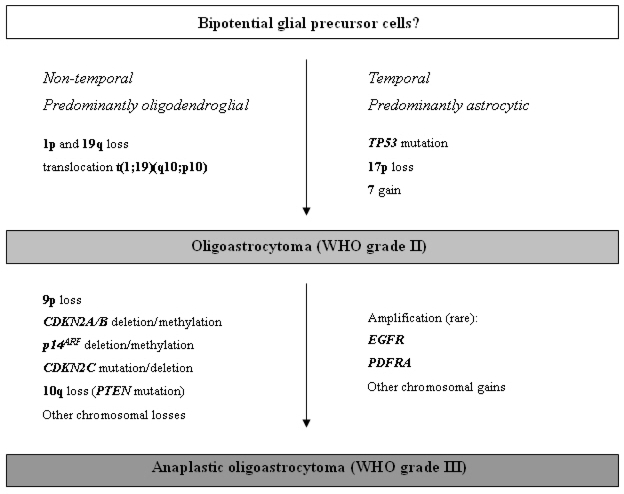
Schematic representation of the molecular pathogenesis and progression of oligoastrocytomas.

Oligoastrocytomas are histologically mixed glial neoplasms with oligodendroglial as well as astrocytic features. The oligodendroglial and astroglial tumor components may either be diffusely intermingled or separated into distinct tumor areas. Genetic alterations that would separate oligoastrocytomas from oligodendrogliomas on the one hand and diffuse astrocytomas on the other hand have not been detected. Roughly half of the oligoastrocytomas show allelic losses on 1p and 19q [71]. While in one study these aberrations were identical in oligodendroglial and astrocytic tumor parts, indicating a monoclonal origin of oligoastrocytomas, other authors reported genetically distinct astrocytic and oligodendroglial components in a subset of oligoastrocytomas [99, 100]. About 30% of oligoastrocytomas carry genetic aberrations typical for diffuse astrocytomas, i.e. *TP53* mutation and loss of heterozygosity on 17p [71]. Those oligoastrocytomas with *TP53* mutation and 17p loss do not have LOH on 1p and 19q, and vice versa. Taken together, these data indicate that oligoastrocytomas are genetically heterogeneous with one subset being genetically related to oligodendrogliomas and another subset corresponding genetically to diffuse astrocytomas (Figure 4).

Oligoastrocytomas that are histologically highly anaplastic and contain areas of necrosis are referred to as “*glioblastomas with oligodendroglial component, WHO grade IV*”. While rare in classic glioblastomas (less than 10% of the cases), 1p/19q deletions may be more common in glioblastomas with an oligodendroglial component, which in part may account for the better survival associated with this particular glioblastoma subgroup [101].

### 2.4. Gliomas with ependymal differentiation

#### 2.4.1. Ependymoma (WHO grade II)

**Figure 5.**
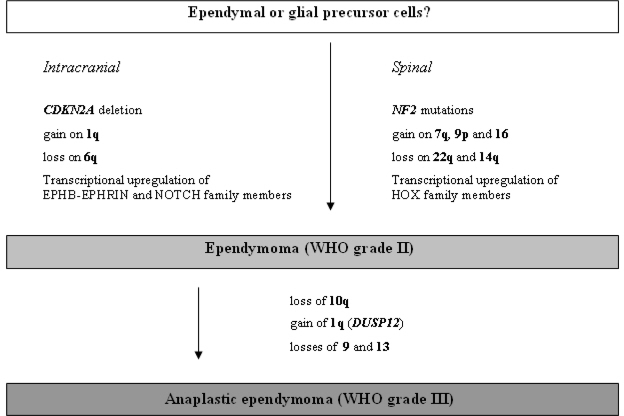
Schematic representation of the molecular pathogenesis and progression of ependymal gliomas.

The most common cytogenetic aberrations in ependymomas are losses of chromosomes 6q, 10, 13, 14, and 22q, as well as gains of chromosomes 1q, 7, 9, 12q, 15q, and 18. Recent studies using CGH analysis reported on distinct patterns of chromosomal aberrations being linked to certain clinical and pathological features of ependymomas, such as patient age, tumor location, and histological subtype or WHO grade [102–106]. Losses on 22q and gains of chromosome 4 were more common in adult tumors [105]. Gains on 1q correlated with the presence of structural chromosomal aberrations, pediatric age, high-grade histology and aggressive clinical behavior [102, 103, 105]. While spinal intramedullary ependymomas preferentially demonstrated losses of chromosomes 22q and 14q as well as gains on chromosomes 7q, 9p and 16, intracranial ependymomas frequently carried gains of 1q and losses on 6q (Figure 5). The distinct genetic profiles associated with tumor location were also reflected in regionally different mRNA expression signatures. Supratentorial ependymomas, for example, expressed elevated levels of members of the EPHB-EPHRIN and NOTCH families, whereas spinal ependymomas showed up-regulated expression of multiple HOX gene family members [106].

Molecular genetic studies on selected candidate genes revealed frequent *NF2* gene mutations in intramedullary spinal ependymomas, while deletions of the *CDKN2A* gene were frequent in intracranial supratentorial ependymomas but rare in ependymomas from other locations [106] (Figure 5). Mutations in the *TP53*, *PTEN* and *INI1* tumor suppressor genes are not a common feature in ependymomas [107–109]. The tumors suppressor genes *RASSF1*, *CDKN2A*, *CDKN2B*, *p14^ARF^* and *TP73*, as well as other genes potentially involved in the tumorigenesis of ependymomas, such as *CASP1*, *MGMT*, *TIMP3* and *THBS1* are epigenetically silenced by aberrant promoter methylation in subsets of ependymomas [110, 111]. As in other gliomas, growth factor receptors, such as EGFR and the related ERBB2 and ERBB4 receptors, are commonly upregulated in ependymal tumors; amplifications of the respective genes, however, do not usually occur in low-grade ependymomas [105, 112].

#### 2.4.2. Anaplastic ependymoma (WHO grade III)

The molecular changes observed in anaplastic ependymomas are fairly similar to those observed in their low-grade counterparts. Though to a certain extent malignant progression in ependymomas may occur, only few molecular changes have been pinpointed that are more or less exclusively associated with a high-grade ependymoma phenotype. In an analysis of 23 anaplastic ependymomas, loss of chromosome arm 10q appeared overrepresented [107]. Also, gain of 1q, with *DUSP12* on 1q23 as a potential target gene [105], as well as losses of 9 and 13 were associated with WHO grade III lesions [113] (Figure 5). In regard to tumor location, mRNA expression analysis distinguished between supratentorial WHO grade II and III tumors. In contrast, infratentorial ependymomas could not that easily be graded by their mRNA expression profiles, suggesting that in this location WHO grade III tumors differ by relatively few genetic changes from WHO grade II tumors [114].

#### 2.4.3. Myxopapillary ependymoma (WHO grade I)

In spite of their benign clinical behavior, myxopapillary ependymomas are often aneuploid or tetraploid and carry numerous chromosomal imbalances, as determined by CGH analysis. In fact, the average number of chromosomal aberrations per tumor is considerably higher than that in ependymomas and anaplastic ependymomas [115]. The most common imbalances are concurrent gains of chromosomes 9 and 18 [116]. Additional recurrent alterations include gains of chromosomes 3, 4, 7, 8, 11, 13, 17q, 20 and X, as well as losses of chromosomes 10 and 22. Also, cDNA profiles with high expression levels of HOXB5, PLA2G5 and ITH2 in myxopapillary ependymomas clearly differed from those in intracranial ependymomas [114], thus indicating that myxopapillary ependymomas are molecularly distinct from other ependymal tumors.

#### 2.4.4. Subependymoma (WHO grade I)

Molecular genetic data on subependymomas are scarce. Cytogenetic investigation of two tumors revealed no structural or numerical abnormalities [117]. Individual cases studied for allelic losses on chromosome arms 10q and 22q, as well as for mutations in the *hSNF5/INI1*, *NF2* and *PTEN* genes also did not show any aberrations [117].

## 3. Prognostic and predictive relevance of molecular changes

A prognostic factor is any measurement available at the time of surgery that correlates with disease-free or overall survival in the absence of systemic adjuvant therapy and thus conveys information on the natural course of the disease. In contrast, a predictive factor is any measurement associated with response to a given therapy.

Many of the above described molecular alterations have been investigated in regard to their prognostic and/or predictive implications, but only few qualified as clinically relevant biomarkers. In this regard, the undisputably most important molecular alterations are hypermethylation of the *MGMT* gene in glioblastomas (Breakout Box 1) and combined deletions of chromosome arms 1p and 19q in oligodendrogliomas (Breakout Box 2). *MGMT* hypermethylation has been clearly identified as a *predictive* marker for the response of glioblastomas to alkylating chemotherapy with nitrosourea [33], temozolomide [34], or a combination of both [35]. The question of whether combined 1p/19q loss in oligodendroglial tumors, in addition to its predictive value for response to radiotherapy and chemotherapy with PCV, can also be regarded as a prognostic biomarker is controversially discussed. In the initial publication by Cairncross and colleagues in 1998 as well as in several retrospective follow-up studies, combined 1p/19q loss correlated with prolonged survival in patients with high-grade oligodendroglial tumors [3, 4, 87, 88, 91–93]. However, as patients with anaplastic oligodendrogliomas receive either radiotherapy or chemotherapy, or a combination of both after surgery as a standard of care, the prognostic value of combined 1p/19q loss cannot be evaluated independently of the administration of adjuvant treatment in high-grade oligodendroglioma patients. Several studies on low-grade WHO grade II oligodendroglioma patients who were initially treated with surgery alone reported an association of 1p/19q deletions with a trend towards longer survival [92–95, 118]. Nevertheless, contrary findings have also been reported, which could not corroborate combined 1p/19q loss as a sensitive prognostic biomarker in patients with oligodendroglial tumors who did not receive radiotherapy or chemotherapy [98].

While the molecular mechanism by which the MGMT repair protein counteracts the therapeutic effects of alkylating chemotherapy and thereby leads to treatment failure is obvious, the mechanism underlying the association of the 1p/19q allelic status with therapy response and longer survival still remains enigmatic. As outlined above, the relevant genes on 1p and 19q are still unidentified and it is unclear whether alterations in one or more genes on these chromosome arms, or rather in completely different chromosomal locations, may account for the clinically less aggressive behavior of 1p/19q-deleted tumors. The observation that *MGMT* promoter hypermethylation is common in oligodendrogliomas with losses on 1p and 19q may point to at least one possible mechanism contributing to the chemosensitivity of these tumors [85]. Furthermore, one may speculate that not primarily the presence of 1p/19q loss but rather the absence of other prognostically unfavorable genetic alterations in 1p/19q-deleted tumors, e.g. losses of chromosome arms 9p, 10 and 18q or gains of chromosomes 7, 8q, 19q and 20, are responsible for the distinct clinical behavior [79]. All these unresolved issues will be subject to future molecular and translational studies.

## 4. Molecular genetics and glioma classification

To date, gliomas are classified based on histological criteria according to the WHO Classification of Tumors of the Central Nervous System in its newest 2007 edition [2]. While immunohistochemistry using antibodies against a number of distinct antigens may support the histological classification, only few of the above described genetic alterations are consistently enough associated with defined glioma subtypes to qualify as auxiliary molecular diagnostic markers.

Analysis for losses on 1p and 19q as well as mutations of the *TP53* gene might help in the differential diagnosis between oligodendroglial and astrocytic gliomas. While oligodendrogliomas exhibit deletions on 1p and 19q in about two-thirds of cases, mutations of the *TP53* gene are a hallmark genetic alteration found in about 60% of diffusely infiltrating astrocytic gliomas. Thus, the demonstration of allelic losses on 1p and 19q argues in favor of an oligodendroglioma, whereas *TP53* mutation or the immunohistochemical detection of a nuclear accumulation of the p53 protein supports the diagnosis of a diffuse astrocytoma. However, the sensitivity of both molecular alterations as differential diagnostic markers is not very high, indicated by the fact that about one third of oligodendrogliomas retain 1p/19 and about 40% of diffuse astrocytomas lack *TP53* mutations and nuclear p53 accumulation. Consequently, 1p/19q testing cannot serve as a decisive diagnostic criterion to identify oligodendrogliomas, or in other words, it is not recommended to “rule in” or “rule out” a diagnosis of oligodendroglioma [71]. In line with this statement, the definition of oligodendroglioma in the latest WHO classification recognizes the frequent presence of 1p/19q deletions in these tumors but does not require this genetic alteration as an obligatory feature for making the diagnosis of oligodendroglioma.

Another diagnostic problem that may prospectively be moderated by help of molecular markers is the differential diagnosis between diffuse and pilocytic astrocytomas. In addition to frequent *TP53* mutations, a recent integrated genomic analysis identified the isocitrate dehydrogenase 1 (*IDH1*) gene as frequently mutated in diffusely infiltrating astrocytic gliomas. Interestingly, in an initial screen on 22 glioblastoma patients, mutations in the active site of *IDH1* occurred in a large fraction of young patients and were associated with an increased overall survival in secondary glioblastoma patients [10]. A follow-up study analyzed *IDH1* codon 132 mutations in a large series of 685 brain tumors comprising all major glioma subtypes and reported *IDH1* mutation frequencies of up to 70% in diffuse astrocytomas, while virtually no mutations were detected in pilocytic astrocytomas [9]. In contrast, pilocytic astrocytomas -as recently indicated- are molecularly characterized by gene duplication/fusion or mutation of the *BRAF* gene on 7q34. These *BRAF* gene alterations occur in about 60–80% of pilocytic astrocytomas but are infrequent in diffusely infiltrating low-grade astrocytomas [46, 51]. Of note, this finding may not only bear diagnostic but also potential clinical relevance in regard to the development of novel targeted therapies. Tumors with duplications or activating mutations of the *BRAF* proto-oncogene showed significantly increased mRNA levels of *BRAF* and its downstream target, *CCND1*, as compared to tumors without these molecular alterations. In subsequent functional analyses both the stable silencing of *BRAF* through shRNA lentiviral transduction and pharmacological inhibition of MEK1/2, the immediate downstream phosphorylation target of *BRAF*, blocked the proliferation and arrested the growth of cultured tumor cells derived from low-grade gliomas [46]. Thus, pharmacological inhibition of the MAPK pathway may serve as a potential treatment option in pediatric low-grade astrocytoma patients.

## 5. Comprehensive molecular analysis of gliomas by means of high-throughput profiling techniques

The genome-wide search for new tumor suppressor genes and oncogenes as well as more sophisticated tumor-specific genomic or transcriptional signatures has been enormously facilitated by the development of novel array-based profiling techniques that nowadays are available for studies at the transcriptional as well as the genomic and epigenetic level. Recent studies indicate that array-based profiling techniques may serve as a valuable adjunct to mere histological classification and may facilitate diagnosis especially in tumors that exhibit borderline histopathological features.

Genomic profiling by means of array-CGH closely paralleled histological classification and proved a powerful technique allowing for an automated genomic profiling of gliomas [119]. Microarray-based gene expression profiling was capable of classifying diagnostically challenging malignant gliomas in a manner that better correlated with clinical outcome than did standard pathology [120]. Moreover, gene expression profiling turned out to be an even more powerful survival predictor than histological grade or patient age [121]. While the standard implementation of whole-genome expression chips in routine diagnostics is not realistic due to its lack of cost-effectiveness, microarray-derived diagnostic and prognostic signatures comprising a restricted number of highly distinctive transcripts reliably classified gliomas into histologically unrecognized biological and prognostic groups [121–123]. Thus, microarray analysis has the potential to unveil confined expression signatures that in a routine diagnostic setting can identify clinically relevant patient subsets.

**Figure 6.**
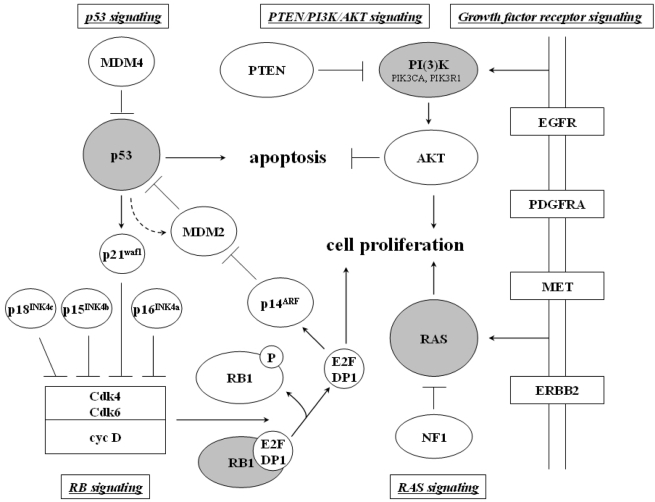
Core pathways involved in the pathogenesis of gliomas. Note the interrelationships between p53, RB, growth factor receptor, PTEN/PI3K/AKT and RAS signaling on the regulation of cell proliferation and apoptosis. While *TP53* mutation, amplification of *MDM2/MDM4* or *p14^ARF^* deletion/methylation inhibits apoptosis, alterations in p16^INK4a^, p15^INK4b^, p18^INK4c^ and p21^waf1^ disinhibit cell cycle progression at the G_1_/S-phase checkpoint via cyclin-dependent kinases by phosphorylation of RB1 and release of E2F transcription factors. Amplification, overexpression or mutation of growth factor receptors stimulates cell proliferation and inhibits apoptosis through both the RAS as well as the PI3K/AKT signaling pathway. The RAS signaling pathway can be alternatively activated by mutations in the *NF1*, the PI3K/AKT signaling pathway by mutations in the *PTEN* gene and less commonly, the *PIK3CA* or the *PIK3R1* gene.

Recent milestones are the publications of two large-scale multi-dimensional studies, one by Johns Hopkins researchers in *Science* [10], the other by The Cancer Genome Atlas Research Network in *Nature* [124]. Parsons and colleagues investigated 22 human glioblastoma samples for genome-wide DNA copy number and gene expression aberrations as well as somatic mutations in 20,661 protein coding genes. In addition to the identification of yet unknown *IDH1* mutations, integrative data analysis identified a set of glioblastoma candidiate cancer genes that mainly functioned within the TP53, RB1 and PI3K/PTEN signaling pathways [10] (Figure 6).

Integrative analysis of DNA copy number, gene expression and DNA methylation profiling in 206 human glioblastomas by The Cancer Genome Atlas Research Network came to fairly similar results. The important pathways, each of which was found to be disrupted in more than three-quarters of glioblastomas were the CDK/cyclin/CDK inhibitor/RB pathway, which is involved in the regulation of cell division; the p53 pathway, which is involved in response to DNA damage and cell death; and the RTK/RAS/PI3K pathway, which is involved in the regulation of growth factor signals (Figure 6). Large-scale sequencing in a subset of 91 human glioblastomas for mutations in 601 selected genes additionally revealed *ERBB2* gene mutations in 8% of glioblastomas, *NF1* gene mutations in 14% and *PIK3R1* mutations in closely 10% of glioblastomas, i.e. at slightly higher incidences as noted before [124–126].

These molecular alterations may impact future treatment strategies. For example, as *PIK3R1* encodes the regulatory protein p85a subunit, response to PI3K inhibitors may depend on whether the tumors bear mutations in this specific gene or not. Also, in regard to predicting sensitivity and the development of resistence to temozolomide the Cancer Genome Atlas Research Network added further support for a role of the DNA mismatch repair system. *MGMT* methylation in conjunction with temozolomide treatment may lead to a loss of mismatch repair function by introduction of mutations in mismatch repair genes [127, 128]. Thus, patients who initially responded to frontline therapy may evolve treatment resistence by developing a hypermutator phenotype. As a consequence, selective strategies targeting mismatch-repair-deficient cells would represent a rational upfront combination with alkylating agents to prevent or minimize resistence to temozolomide [127]. A further recent study aiming at the identification of molecular profiles specific for treatment resistance to temozolomide identified a “glioma stem cell” or “self-renewal” expression signature as a predictor of poor survival [129]. Dominated by HOX genes and containing the putative glioma stem cell marker Prominin-1 (CD133), this signature proved an independent prognostic factor also in multivariate analysis, adjusted for *MGMT* methylation status. Thus, high-throughput profiling approaches might help to uncover mechanisms of treatment resistence and could accelerate the generation of therapeutically relevant biomarkers.

## 6. Conclusions

In this review we have summarized the most important cytogenetic and molecular changes associated with the different histological subtypes and grades of gliomas. Though molecular glioma classification is still in its infancy, tremendous advances have been made in regard to the exploitation of molecular changes for complementary diagnostic purposes and the establishment of novel predictive and prognostic biomarkers. High-throughput profiling techniques in conjunction with sophisticated bioinformatic integrative tools are emerging to revolutionize our knowledge about the complexity of the disease. The identification of glioma core pathways will enable us to deal with this complexity, especially in regard to the development of novel efficient targeted therapies.

## Figures and Tables

**Table 1.** Classification and grading of the main glioma subtypes according to the WHO classification of tumors of the central nervous system [2].

Tumor type	WHO grade
*Diffusely infiltrating astrocytic gliomas*	
**Diffuse astrocytoma**	II
**Anaplastic astrocytoma**	III
**Glioblastoma**	IV
Giant cell glioblastoma	IV
Gliosarcoma	IV
*Astrocytic gliomas with more circumscribed growth*	
**Pilocytic astrocytoma**	I
Pilomyxoid astrocytoma	II
**Pleomorphic xanthoastrocytoma**	II
**Subependymal giant cell astrocytoma**	I
*Oligodendrogliomas and mixed gliomas*	
**Oligodendroglioma**	II
**Anaplastic oligodendroglioma**	III
**Oligoastrocytoma**	II
**Anaplastic oligoastrocytoma**	III
*Gliomas with ependymal differentiation*	
**Subependymoma**	I
**Myxopapillary ependymoma**	I
**Ependymoma**	II
**Anaplastic ependymoma**	III

## References

[1] Ohgaki H, Kleihues P (2005). Epidemiology and etiology of gliomas. Acta Neuropathol. (Berl.).

[2] Louis DN, Ohgaki H, Wiestler OD, Cavenee WK (2007). WHO Classification of Tumours of the Central Nervous System.

[3] Cairncross G, Berkey B, Shaw E, Jenkins R, Scheithauer B, Brachman D, Buckner J, Fink K, Souhami L, Laperierre N, Mehta M, Curran W (2006). Phase III trial of chemotherapy plus radiotherapy compared with radiotherapy alone for pure and mixed anaplastic oligodendroglioma: Intergroup Radiation Therapy Oncology Group Trial 9402. J. Clin. Oncol.

[4] van den Bent MJ, Carpentier AF, Brandes AA, Sanson M, Taphoorn MJ, Bernsen HJ, Frenay M, Tijssen CC, Grisold W, Sipos L, Haaxma-Reiche H, Kros JM, van Kouwenhoven MC, Vecht CJ, Allgeier A, Lacombe D, Gorlia T (2006). Adjuvant procarbazine, lomustine, and vincristine improves progression-free survival but not overall survival in newly diagnosed anaplastic oligodendrogliomas and oligoastrocytomas: A randomized European Organisation for Research and Treatment of Cancer phase III trial. J. Clin. Oncol.

[5] Nishizaki T, Ozaki S, Harada K, Ito H, Arai H, Beppu T, Sasaki K (1998). Investigation of genetic alterations associated with the grade of astrocytic tumor by comparative genomic hybridization. Genes Chromosom. Cancer.

[6] Schrock E, Blume C, Meffert MC, du Manoir S, Bersch W, Kiessling M, Lozanowa T, Thiel G, Witkowski R, Ried T, Cremer T (1996). Recurrent gain of chromosome arm 7q in low-grade astrocytic tumors studied by comparative genomic hybridization. Genes Chromosom. Cancer.

[7] Reifenberger G, Collins VP (2004). Pathology and molecular genetics of astrocytic gliomas. J. Mol. Med.

[8] Ichimura K, Bolin MB, Goike HM, Schmidt EE, Moshref A, Collins VP (2000). Deregulation of the p14ARF/MDM2/p53 pathway is a prerequisite for human astrocytic gliomas with G1-S transition control gene abnormalities. Cancer Res.

[9] Balss J, Meyer J, Mueller W, Korshunov A, Hartmann C, von Deimling A (2008). Analysis of the IDH1 codon 132 mutation in brain tumors. Acta Neuropathol.

[10] Parsons DW, Jones S, Zhang X, Lin JC, Leary RJ, Angenendt P, Mankoo P, Carter H, Siu IM, Gallia GL, Olivi A, McLendon R, Rasheed BA, Keir S, Nikolskaya T, Nikolsky Y, Busam DA, Tekleab H, Diaz LA, Hartigan J, Smith DR, Strausberg RL, Marie SK, Shinjo SM, Yan H, Riggins GJ, Bigner DD, Karchin R, Papadopoulos N, Parmigiani G, Vogelstein B, Velculescu VE, Kinzler KW (2008). An integrated genomic analysis of human glioblastoma multiforme. Science.

[11] Watanabe K, Peraud A, Gratas C, Wakai S, Kleihues P, Ohgaki H (1998). p53 and PTEN gene mutations in gemistocytic astrocytomas. Acta Neuropathol. (Berl.).

[12] Watanabe T, Katayama Y, Yoshino A, Yachi K, Ohta T, Ogino A, Komine C, Fukushima T (2007). Aberrant hypermethylation of p14ARF and O6-methylguanine-DNA methyltransferase genes in astrocytoma progression. Brain Pathol.

[13] Waha A, Guntner S, Huang TH, Yan PS, Arslan B, Pietsch T, Wiestler OD (2005). Epigenetic silencing of the protocadherin family member PCDH-gamma-A11 in astrocytomas. Neoplasia.

[14] Kunitz A, Wolter M, van den Boom J, Felsberg J, Tews B, Hahn M, Benner A, Sabel M, Lichter P, Reifenberger G, von Deimling A, Hartmann C (2007). DNA hypermethylation and Aberrant Expression of the EMP3 Gene at 19q13.3 in Human Gliomas. Brain Pathol.

[15] Hermanson M, Funa K, Hartman M, Claesson-Welsh L, Heldin CH, Westermark B, Nister M (1992). Platelet-derived growth factor and its receptors in human glioma tissue: Expression of messenger RNA and protein suggests the presence of autocrine and paracrine loops. Cancer Res.

[16] Fleming TP, Saxena A, Clark WC, Robertson JT, Oldfield EH, Aaronson SA, Ali IU (1992). Amplification and/or overexpression of platelet-derived growth factor receptors and epidermal growth factor receptor in human glial tumors. Cancer Res.

[17] Reifenberger G, Reifenberger J, Ichimura K, Meltzer PS, Collins VP (1994). Amplification of multiple genes from chromosomal region 12q13–14 in human malignant gliomas: preliminary mapping of the amplicons shows preferential involvement of CDK4, SAS, and MDM2. Cancer Res.

[18] Ichimura K, Schmidt EE, Goike HM, Collins VP (1996). Human glioblastomas with no alterations of the CDKN2A (p16INK4A, MTS1) and CDK4 genes have frequent mutations of the retinoblastoma gene. Oncogene.

[19] Schmidt EE, Ichimura K, Reifenberger G, Collins VP (1994). CDKN2 (p16/MTS1) gene deletion or CDK4 amplification occurs in the majority of glioblastomas. Cancer Res.

[20] Smith JS, Tachibana I, Passe SM, Huntley BK, Borell TJ, Iturria N, O’Fallon JR, Schaefer PL, Scheithauer BW, James CD, Buckner JC, Jenkins RB (2001). PTEN mutation, EGFR amplification, and outcome in patients with anaplastic astrocytoma and glioblastoma multiforme. J. Natl. Cancer Inst.

[21] Ohgaki H, Kleihues P (2007). Genetic pathways to primary and secondary glioblastoma. Am. J. Pathol.

[22] Nakamura M, Watanabe T, Klangby U, Asker C, Wiman K, Yonekawa Y, Kleihues P, Ohgaki H (2001). p14ARF deletion and methylation in genetic pathways to glioblastomas. Brain Pathol.

[23] Reifenberger G, Ichimura K, Reifenberger J, Elkahloun AG, Meltzer PS, Collins VP (1996). Refined mapping of 12q13–q15 amplicons in human malignant gliomas suggests CDK4/SAS and MDM2 as independent amplification targets. Cancer Res.

[24] Riemenschneider MJ, Buschges R, Wolter M, Reifenberger J, Bostrom J, Kraus JA, Schlegel U, Reifenberger G (1999). Amplification and overexpression of the MDM4 (MDMX) gene from 1q32 in a subset of malignant gliomas without TP53 mutation or MDM2 amplification. Cancer Res.

[25] Riemenschneider MJ, Knobbe CB, Reifenberger G (2003). Refined mapping of 1q32 amplicons in malignant gliomas confirms MDM4 as the main amplification target. Int. J. Cancer.

[26] Burger PC, Pearl DK, Aldape K, Yates AJ, Scheithauer BW, Passe SM, Jenkins RB, James CD (2001). Small cell architecture--a histological equivalent of EGFR amplification in glioblastoma multiforme?. J. Neuropathol. Exp. Neurol.

[27] Nakamura M, Yang F, Fujisawa H, Yonekawa Y, Kleihues P, Ohgaki H (2000). Loss of heterozygosity on chromosome 19 in secondary glioblastomas. J. Neuropathol. Exp. Neurol.

[28] Tepel M, Roerig P, Wolter M, Gutmann DH, Perry A, Reifenberger G, Riemenschneider MJ (2008). Frequent promoter hypermethylation and transcriptional downregulation of the NDRG2 gene at 14q11.2 in primary glioblastoma. Int. J. Cancer.

[29] Mueller W, Nutt CL, Ehrich M, Riemenschneider MJ, von Deimling A, van den Boom D, Louis DN (2007). Downregulation of RUNX3 and TES by hypermethylation in glioblastoma. Oncogene.

[30] Riemenschneider MJ, Betensky RA, Pasedag SM, Louis DN (2006). AKT activation in human glioblastomas enhances proliferation via TSC2 and S6 kinase signaling. Cancer Res.

[31] Riemenschneider MJ, Mueller W, Betensky RA, Mohapatra G, Louis DN (2005). *In situ* analysis of integrin and growth factor receptor signaling pathways in human glioblastomas suggests overlapping relationships with focal adhesion kinase activation. Am. J. Pathol.

[32] Gerson SL (2004). MGMT: Its role in cancer aetiology and cancer therapeutics. Nat. Rev. Cancer.

[33] Esteller M, Garcia-Foncillas J, Andion E, Goodman SN, Hidalgo OF, Vanaclocha V, Baylin SB, Herman JG (2000). Inactivation of the DNA-repair gene MGMT and the clinical response of gliomas to alkylating agents. N. Engl. J. Med.

[34] Hegi ME, Diserens AC, Gorlia T, Hamou MF, de Tribolet N, Weller M, Kros JM, Hainfellner JA, Mason W, Mariani L, Bromberg JE, Hau P, Mirimanoff RO, Cairncross JG, Janzer RC, Stupp R (2005). MGMT gene silencing and benefit from temozolomide in glioblastoma. N. Engl. J. Med.

[35] Herrlinger U, Rieger J, Koch D, Loeser S, Blaschke B, Kortmann RD, Steinbach JP, Hundsberger T, Wick W, Meyermann R, Tan TC, Sommer C, Bamberg M, Reifenberger G, Weller M (2006). Phase II trial of lomustine plus temozolomide chemotherapy in addition to radiotherapy in newly diagnosed glioblastoma: UKT-03. J. Clin. Oncol.

[36] Meyer-Puttlitz B, Hayashi Y, Waha A, Rollbrocker B, Bostrom J, Wiestler OD, Louis DN, Reifenberger G, von Deimling A (1997). Molecular genetic analysis of giant cell glioblastomas. Am. J. Pathol.

[37] Peraud A, Watanabe K, Schwechheimer K, Yonekawa Y, Kleihues P, Ohgaki H (1999). Genetic profile of the giant cell glioblastoma. Lab. Invest.

[38] Reis RM, Konu-Lebleblicioglu D, Lopes JM, Kleihues P, Ohgaki H (2000). Genetic profile of gliosarcomas. Am. J. Pathol.

[39] Actor B, Cobbers JM, Buschges R, Wolter M, Knobbe CB, Lichter P, Reifenberger G, Weber RG (2002). Comprehensive analysis of genomic alterations in gliosarcoma and its two tissue components. Genes Chromosom. Cancer.

[40] Rickert CH, Paulus W (2002). No chromosomal imbalances detected by comparative genomic hybridisation in subependymal giant cell astrocytomas. Acta Neuropathol. (Berl.).

[41] Giangaspero F, Kaulich K, Cenacchi G, Cerasoli S, Lerch KD, Breu H, Reuter T, Reifenberger G (2002). Lipoastrocytoma: a rare low-grade astrocytoma variant of pediatric age. Acta Neuropathol.

[42] Kepes JJ, Fulling KH, Garcia JH (1982). The clinical significance of “adenoid” formations of neoplastic astrocytes, imitating metastatic carcinoma, in gliosarcomas. A review of five cases. Clin. Neuropathol.

[43] Mathews T, Moossy J (1974). Gliomas containing bone and cartilage. J. Neuropathol. Exp. Neurol.

[44] Mork SJ, Rubinstein LJ, Kepes JJ, Perentes E, Uphoff DF (1988). Patterns of epithelial metaplasia in malignant gliomas. II. Squamous differentiation of epithelial-like formations in gliosarcomas and glioblastomas. J. Neuropathol. Exp. Neurol.

[45] Sanoudou D, Tingby O, Ferguson-Smith MA, Collins VP, Coleman N (2000). Analysis of pilocytic astrocytoma by comparative genomic hybridization. Br. J. Cancer.

[46] Pfister S, Janzarik WG, Remke M, Ernst A, Werft W, Becker N, Toedt G, Wittmann A, Kratz C, Olbrich H, Ahmadi R, Thieme B, Joos S, Radlwimmer B, Kulozik A, Pietsch T, Herold-Mende C, Gnekow A, Reifenberger G, Korshunov A, Scheurlen W, Omran H, Lichter P (2008). BRAF gene duplication constitutes a mechanism of MAPK pathway activation in low-grade astrocytomas. J. Clin. Invest.

[47] Kluwe L, Hagel C, Tatagiba M, Thomas S, Stavrou D, Ostertag H, von Deimling A, Mautner VF (2001). Loss of NF1 alleles distinguish sporadic from NF1-associated pilocytic astrocytomas. J. Neuropathol. Exp. Neurol.

[48] Wimmer K, Eckart M, Meyer-Puttlitz B, Fonatsch C, Pietsch T (2002). Mutational and expression analysis of the NF1 gene argues against a role as tumor suppressor in sporadic pilocytic astrocytomas. J. Neuropathol. Exp. Neurol.

[49] Lang FF, Miller DC, Pisharody S, Koslow M, Newcomb EW (1994). High frequency of p53 protein accumulation without p53 gene mutation in human juvenile pilocytic, low grade and anaplastic astrocytomas. Oncogene.

[50] Ohgaki H, Eibl RH, Schwab M, Reichel MB, Mariani L, Gehring M, Petersen I, Holl T, Wiestler OD, Kleihues P (1993). Mutations of the p53 tumor suppressor gene in neoplasms of the human nervous system. Mol. Carcinog.

[51] Jones DT, Kocialkowski S, Liu L, Pearson DM, Backlund LM, Ichimura K, Collins VP (2008). Tandem duplication producing a novel oncogenic BRAF fusion gene defines the majority of pilocytic astrocytomas. Cancer Res.

[52] Bar EE, Lin A, Tihan T, Burger PC, Eberhart CG (2008). Frequent gains at chromosome 7q34 involving BRAF in pilocytic astrocytoma. J. Neuropathol. Exp. Neurol.

[53] Deshmukh H, Yeh TH, Yu J, Sharma MK, Perry A, Leonard JR, Watson MA, Gutmann DH, Nagarajan R (2008). High-resolution, dual-platform aCGH analysis reveals frequent HIPK2 amplification and increased expression in pilocytic astrocytomas. Oncogene.

[54] Tihan T, Fisher PG, Kepner JL, Godfraind C, McComb RD, Goldthwaite PT, Burger PC (1999). Pediatric astrocytomas with monomorphous pilomyxoid features and a less favorable outcome. J. Neuropathol. Exp. Neurol.

[55] Rodriguez FJ, Giannini C, Asmann YW, Sharma MK, Perry A, Tibbetts KM, Jenkins RB, Scheithauer BW, Anant S, Jenkins S, Eberhart CG, Sarkaria JN, Gutmann DH (2008). Gene expression profiling of NF-1-associated and sporadic pilocytic astrocytoma identifies aldehyde dehydrogenase 1 family member L1 (ALDH1L1) as an underexpressed candidate biomarker in aggressive subtypes. J. Neuropathol. Exp. Neurol.

[56] Weber RG, Hoischen A, Ehrler M, Zipper P, Kaulich K, Blaschke B, Becker AJ, Weber-Mangal S, Jauch A, Radlwimmer B, Schramm J, Wiestler OD, Lichter P, Reifenberger G (2007). Frequent loss of chromosome 9, homozygous CDKN2A/p14(ARF)/CDKN2B deletion and low TSC1 mRNA expression in pleomorphic xanthoastrocytomas. Oncogene.

[57] Giannini C, Hebrink D, Scheithauer BW, Dei Tos AP, James CD (2001). Analysis of p53 mutation and expression in pleomorphic xanthoastrocytoma. Neurogenetics.

[58] Kaulich K, Blaschke B, Numann A, von Deimling A, Wiestler OD, Weber RG, Reifenberger G (2002). Genetic alterations commonly found in diffusely infiltrating cerebral gliomas are rare or absent in pleomorphic xanthoastrocytomas. J. Neuropathol. Exp. Neurol.

[59] Chan JA, Zhang H, Roberts PS, Jozwiak S, Wieslawa G, Lewin-Kowalik J, Kotulska K, Kwiatkowski DJ (2004). Pathogenesis of tuberous sclerosis subependymal giant cell astrocytomas: biallelic inactivation of TSC1 or TSC2 leads to mTOR activation. J. Neuropathol. Exp. Neurol.

[60] Riemenschneider MJ, Koy TH, Reifenberger G (2004). Expression of oligodendrocyte lineage genes in oligodendroglial and astrocytic gliomas. Acta Neuropathol.

[61] Bouvier C, Bartoli C, Aguirre-Cruz L, Virard I, Colin C, Fernandez C, Gouvernet J, Figarella-Branger D (2003). Shared oligodendrocyte lineage gene expression in gliomas and oligodendrocyte progenitor cells. J. Neurosurg.

[62] Ohnishi A, Sawa H, Tsuda M, Sawamura Y, Itoh T, Iwasaki Y, Nagashima K (2003). Expression of the oligodendroglial lineage-associated markers Olig1 and Olig2 in different types of human gliomas. J. Neuropathol. Exp. Neurol.

[63] Reifenberger J, Reifenberger G, Liu L, James CD, Wechsler W, Collins VP (1994). Molecular genetic analysis of oligodendroglial tumors shows preferential allelic deletions on 19q and 1p. Am. J. Pathol.

[64] Griffin CA, Burger P, Morsberger L, Yonescu R, Swierczynski S, Weingart JD, Murphy KM (2006). Identification of der(1;19)(q10;p10) in five oligodendrogliomas suggests mechanism of concurrent 1p and 19q loss. J. Neuropathol. Exp. Neurol.

[65] Jenkins RB, Blair H, Ballman KV, Giannini C, Arusell RM, Law M, Flynn H, Passe S, Felten S, Brown PD, Shaw EG, Buckner JC (2006). A t(1;19)(q10;p10) mediates the combined deletions of 1p and 19q and predicts a better prognosis of patients with oligodendroglioma. Cancer Res.

[66] Boulay JL, Miserez AR, Zweifel C, Sivasankaran B, Kana V, Ghaffari A, Luyken C, Sabel M, Zerrouqi A, Wasner M, Van Meir E, Tolnay M, Reifenberger G, Merlo A (2007). Loss of NOTCH2 positively predicts survival in subgroups of human glial brain tumors. PLoS ONE.

[67] Barbashina V, Salazar P, Holland EC, Rosenblum MK, Ladanyi M (2005). Allelic losses at 1p36 and 19q13 in gliomas: Correlation with histologic classification, definition of a 150-kb minimal deleted region on 1p36, and evaluation of CAMTA1 as a candidate tumor suppressor gene. Clin. Cancer Res.

[68] Dong S, Pang JC, Hu J, Zhou LF, Ng HK (2002). Transcriptional inactivation of TP73 expression in oligodendroglial tumors. Int. J. Cancer.

[69] McDonald JM, Dunlap S, Cogdell D, Dunmire V, Wei Q, Starzinski-Powitz A, Sawaya R, Bruner J, Fuller GN, Aldape K, Zhang W (2006). The SHREW1 gene, frequently deleted in oligodendrogliomas, functions to inhibit cell adhesion and migration. Cancer Biol. Ther.

[70] McDonald JM, Dunmire V, Taylor E, Sawaya R, Bruner J, Fuller GN, Aldape K, Zhang W (2005). Attenuated expression of DFFB is a hallmark of oligodendrogliomas with 1p-allelic loss. Mol. Cancer.

[71] Reifenberger G, Louis DN (2003). Oligodendroglioma: toward molecular definitions in diagnostic neuro-oncology. J. Neuropathol. Exp. Neurol.

[72] Riemenschneider MJ, Reifenberger J, Reifenberger G (2008). Frequent biallelic inactivation and transcriptional silencing of the DIRAS3 gene at 1p31 in oligodendroglial tumors with 1p loss. Int. J. Cancer.

[73] Tews B, Roerig P, Hartmann C, Hahn M, Felsberg J, Blaschke B, Sabel M, Kunitz A, Toedt G, Neben K, Benner A, Deimling A, Reifenberger G, Lichter P (2007). Hypermethylation and transcriptional downregulation of the CITED4 gene at 1p34.2 in oligodendroglial tumours with allelic losses on 1p and 19q. Oncogene.

[74] Wolf RM, Draghi N, Liang X, Dai C, Uhrbom L, Eklof C, Westermark B, Holland EC, Resh MD (2003). p190RhoGAP can act to inhibit PDGF-induced gliomas in mice: a putative tumor suppressor encoded on human chromosome 19q13.3. Genes Dev.

[75] Alaminos M, Davalos V, Ropero S, Setien F, Paz MF, Herranz M, Fraga MF, Mora J, Cheung NK, Gerald WL, Esteller M (2005). EMP3, a myelin-related gene located in the critical 19q13.3 region, is epigenetically silenced and exhibits features of a candidate tumor suppressor in glioma and neuroblastoma. Cancer Res.

[76] Hong C, Bollen AW, Costello JF (2003). The contribution of genetic and epigenetic mechanisms to gene silencing in oligodendrogliomas. Cancer Res.

[77] Trouillard O, Aguirre-Cruz L, Hoang-Xuan K, Marie Y, Delattre JY, Sanson M (2004). Parental 19q loss and PEG3 expression in oligodendrogliomas. Cancer Genet. Cytogenet.

[78] Jeuken JW, von Deimling A, Wesseling P (2004). Molecular pathogenesis of oligodendroglial tumors. J. Neurooncol.

[79] Trost D, Ehrler M, Fimmers R, Felsberg J, Sabel MC, Kirsch L, Schramm J, Wiestler OD, Reifenberger G, Weber RG (2007). Identification of genomic aberrations associated with shorter overall survival in patients with oligodendroglial tumors. Int. J. Cancer.

[80] Watanabe T, Nakamura M, Yonekawa Y, Kleihues P, Ohgaki H (2001). Promoter hypermethylation and homozygous deletion of the p14ARF and p16INK4a genes in oligodendrogliomas. Acta Neuropathol.

[81] Wolter M, Reifenberger J, Blaschke B, Ichimura K, Schmidt EE, Collins VP, Reifenberger G (2001). Oligodendroglial tumors frequently demonstrate hypermethylation of the CDKN2A (MTS1, p16INK4a), p14ARF, and CDKN2B (MTS2, p15INK4b) tumor suppressor genes. J. Neuropathol. Exp. Neurol.

[82] Alonso ME, Bello MJ, Gonzalez-Gomez P, Arjona D, Lomas J, de Campos JM, Isla A, Sarasa JL, Rey JA (2003). Aberrant promoter methylation of multiple genes in oligodendrogliomas and ependymomas. Cancer Genet. Cytogenet.

[83] Dong SM, Pang JC, Poon WS, Hu J, To KF, Chang AR, Ng HK (2001). Concurrent hypermethylation of multiple genes is associated with grade of oligodendroglial tumors. J. Neuropathol. Exp. Neurol.

[84] McLendon RE, Herndon JE, West B, Reardon D, Wiltshire R, Rasheed BK, Quinn J, Friedman HS, Friedman AH, Bigner DD (2005). Survival analysis of presumptive prognostic markers among oligodendrogliomas. Cancer.

[85] Mollemann M, Wolter M, Felsberg J, Collins VP, Reifenberger G (2005). Frequent promoter hypermethylation and low expression of the MGMT gene in oligodendroglial tumors. Int. J. Cancer.

[86] Di Rocco F, Carroll RS, Zhang J, Black PM (1998). Platelet-derived growth factor and its receptor expression in human oligodendrogliomas. Neurosurgery.

[87] Fallon KB, Palmer CA, Roth KA, Nabors LB, Wang W, Carpenter M, Banerjee R, Forsyth P, Rich K, Perry A (2004). Prognostic value of 1p, 19q, 9p, 10q, and EGFR-FISH analyses in recurrent oligodendrogliomas. J. Neuropathol. Exp. Neurol.

[88] Hoang-Xuan K, He J, Huguet S, Mokhtari K, Marie Y, Kujas M, Leuraud P, Capelle L, Delattre JY, Poirier J, Broet P, Sanson M (2001). Molecular heterogeneity of oligodendrogliomas suggests alternative pathways in tumor progression. Neurology.

[89] Sasaki H, Zlatescu MC, Betensky RA, Ino Y, Cairncross JG, Louis DN (2001). PTEN is a target of chromosome 10q loss in anaplastic oligodendrogliomas and PTEN alterations are associated with poor prognosis. Am. J. Pathol.

[90] Broderick DK, Di C, Parrett TJ, Samuels YR, Cummins JM, McLendon RE, Fults DW, Velculescu VE, Bigner DD, Yan H (2004). Mutations of PIK3CA in anaplastic oligodendrogliomas, high-grade astrocytomas, and medulloblastomas. Cancer Res.

[91] Cairncross JG, Ueki K, Zlatescu MC, Lisle DK, Finkelstein DM, Hammond RR, Silver JS, Stark PC, Macdonald DR, Ino Y, Ramsay DA, Louis DN (1998). Specific genetic predictors of chemotherapeutic response and survival in patients with anaplastic oligodendrogliomas. J. Natl. Cancer Inst.

[92] Smith JS, Perry A, Borell TJ, Lee HK, O’Fallon J, Hosek SM, Kimmel D, Yates A, Burger PC, Scheithauer BW, Jenkins RB (2000). Alterations of chromosome arms 1p and 19q as predictors of survival in oligodendrogliomas, astrocytomas, and mixed oligoastrocytomas. J. Clin. Oncol.

[93] Felsberg J, Erkwoh A, Sabel MC, Kirsch L, Fimmers R, Blaschke B, Schlegel U, Schramm J, Wiestler OD, Reifenberger G (2004). Oligodendroglial tumors: Refinement of candidate regions on chromosome arm 1p and correlation of 1p/19q status with survival. Brain Pathol.

[94] Kanner AA, Staugaitis SM, Castilla EA, Chernova O, Prayson RA, Vogelbaum MA, Stevens G, Peereboom D, Suh J, Lee SY, Tubbs RR, Barnett GH (2006). The impact of genotype on outcome in oligodendroglioma: validation of the loss of chromosome arm 1p as an important factor in clinical decision making. J. Neurosurg.

[95] Kujas M, Lejeune J, Benouaich-Amiel A, Criniere E, Laigle-Donadey F, Marie Y, Mokhtari K, Polivka M, Bernier M, Chretien F, Couvelard A, Capelle L, Duffau H, Cornu P, Broet P, Thillet J, Carpentier AF, Sanson M, Hoang-Xuan K, Delattre JY (2005). Chromosome 1p loss: a favorable prognostic factor in low-grade gliomas. Ann. Neurol.

[96] Hoang-Xuan K, Capelle L, Kujas M, Taillibert S, Duffau H, Lejeune J, Polivka M, Criniere E, Marie Y, Mokhtari K, Carpentier AF, Laigle F, Simon JM, Cornu P, Broet P, Sanson M, Delattre JY (2004). Temozolomide as initial treatment for adults with low-grade oligodendrogliomas or oligoastrocytomas and correlation with chromosome 1p deletions. J. Clin. Oncol.

[97] Levin N, Lavon I, Zelikovitsh B, Fuchs D, Bokstein F, Fellig Y, Siegal T (2006). Progressive low-grade oligodendrogliomas: response to temozolomide and correlation between genetic profile and O6-methylguanine DNA methyltransferase protein expression. Cancer.

[98] Weller M, Berger H, Hartmann C, Schramm J, Westphal M, Simon M, Goldbrunner R, Krex D, Steinbach JP, Ostertag CB, Loeffler M, Pietsch T, von Deimling A (2007). Combined 1p/19q loss in oligodendroglial tumors: predictive or prognostic biomarker?. Clin. Cancer Res.

[99] Kraus JA, Koopmann J, Kaskel P, Maintz D, Brandner S, Schramm J, Louis DN, Wiestler OD, von Deimling A (1995). Shared allelic losses on chromosomes 1p and 19q suggest a common origin of oligodendroglioma and oligoastrocytoma. J. Neuropathol. Exp. Neurol.

[100] Qu M, Olofsson T, Sigurdardottir S, You C, Kalimo H, Nister M, Smits A, Ren ZP (2007). Genetically distinct astrocytic and oligodendroglial components in oligoastrocytomas. Acta Neuropathol.

[101] Miller CR, Dunham CP, Scheithauer BW, Perry A (2006). Significance of necrosis in grading of oligodendroglial neoplasms: a clinicopathologic and genetic study of newly diagnosed high-grade gliomas. J. Clin. Oncol.

[102] Carter M, Nicholson J, Ross F, Crolla J, Allibone R, Balaji V, Perry R, Walker D, Gilbertson R, Ellison DW (2002). Genetic abnormalities detected in ependymomas by comparative genomic hybridisation. Br. J. Cancer.

[103] Dyer S, Prebble E, Davison V, Davies P, Ramani P, Ellison D, Grundy R (2002). Genomic imbalances in pediatric intracranial ependymomas define clinically relevant groups. Am. J. Pathol.

[104] Jeuken JW, Sprenger SH, Gilhuis J, Teepen HL, Grotenhuis AJ, Wesseling P (2002). Correlation between localization, age, and chromosomal imbalances in ependymal tumours as detected by CGH. J. Pathol.

[105] Mendrzyk F, Korshunov A, Benner A, Toedt G, Pfister S, Radlwimmer B, Lichter P (2006). Identification of gains on 1q and epidermal growth factor receptor overexpression as independent prognostic markers in intracranial ependymoma. Clin. Cancer Res.

[106] Taylor MD, Poppleton H, Fuller C, Su X, Liu Y, Jensen P, Magdaleno S, Dalton J, Calabrese C, Board J, Macdonald T, Rutka J, Guha A, Gajjar A, Curran T, Gilbertson RJ (2005). Radial glia cells are candidate stem cells of ependymoma. Cancer Cell.

[107] Ebert C, von Haken M, Meyer-Puttlitz B, Wiestler OD, Reifenberger G, Pietsch T, von Deimling A (1999). Molecular genetic analysis of ependymal tumors. NF2 mutations and chromosome 22q loss occur preferentially in intramedullary spinal ependymomas. Am. J. Pathol.

[108] Kraus JA, de Millas W, Sorensen N, Herbold C, Schichor C, Tonn JC, Wiestler OD, von Deimling A, Pietsch T (2001). Indications for a tumor suppressor gene at 22q11 involved in the pathogenesis of ependymal tumors and distinct from hSNF5/INI1. Acta Neuropathol.

[109] Ohgaki H, Eibl RH, Wiestler OD, Yasargil MG, Newcomb EW, Kleihues P (1991). p53 mutations in nonastrocytic human brain tumors. Cancer Res.

[110] Hamilton DW, Lusher ME, Lindsey JC, Ellison DW, Clifford SC (2005). Epigenetic inactivation of the RASSF1A tumour suppressor gene in ependymoma. Cancer Lett.

[111] Rousseau E, Ruchoux MM, Scaravilli F, Chapon F, Vinchon M, De Smet C, Godfraind C, Vikkula M (2003). CDKN2A, CDKN2B and p14ARF are frequently and differentially methylated in ependymal tumours. Neuropathol. Appl. Neurobiol.

[112] Gilbertson RJ, Bentley L, Hernan R, Junttila TT, Frank AJ, Haapasalo H, Connelly M, Wetmore C, Curran T, Elenius K, Ellison DW (2002). ERBB receptor signaling promotes ependymoma cell proliferation and represents a potential novel therapeutic target for this disease. Clin. Cancer Res.

[113] Hirose Y, Aldape K, Bollen A, James CD, Brat D, Lamborn K, Berger M, Feuerstein BG (2001). Chromosomal abnormalities subdivide ependymal tumors into clinically relevant groups. Am. J. Pathol.

[114] Korshunov A, Neben K, Wrobel G, Tews B, Benner A, Hahn M, Golanov A, Lichter P (2003). Gene expression patterns in ependymomas correlate with tumor location, grade, and patient age. Am. J. Pathol.

[115] Scheil S, Bruderlein S, Eicker M, Herms J, Herold-Mende C, Steiner HH, Barth TF, Moller P (2001). Low frequency of chromosomal imbalances in anaplastic ependymomas as detected by comparative genomic hybridization. Brain Pathol.

[116] Mahler-Araujo MB, Sanoudou D, Tingby O, Liu L, Coleman N, Ichimura K, Collins VP (2003). Structural genomic abnormalities of chromosomes 9 and 18 in myxopapillary ependymomas. J. Neuropathol. Exp. Neurol.

[117] Dal Cin P, Van den Berghe H, Buonamici L, Losi L, Roncaroli F, Calbucci F (1999). Cytogenetic investigation in subependymoma. Cancer Genet. Cytogenet.

[118] Sasaki H, Zlatescu MC, Betensky RA, Johnk LB, Cutone AN, Cairncross JG, Louis DN (2002). Histopathological-molecular genetic correlations in referral pathologist-diagnosed low-grade “oligodendroglioma”. J. Neuropathol. Exp. Neurol.

[119] Roerig P, Nessling M, Radlwimmer B, Joos S, Wrobel G, Schwaenen C, Reifenberger G, Lichter P (2005). Molecular classification of human gliomas using matrix-based comparative genomic hybridization. Int. J. Cancer.

[120] Nutt CL, Mani DR, Betensky RA, Tamayo P, Cairncross JG, Ladd C, Pohl U, Hartmann C, McLaughlin ME, Batchelor TT, Black PM, von Deimling A, Pomeroy SL, Golub TR, Louis DN (2003). Gene expression-based classification of malignant gliomas correlates better with survival than histological classification. Cancer Res.

[121] Freije WA, Castro-Vargas FE, Fang Z, Horvath S, Cloughesy T, Liau LM, Mischel PS, Nelson SF (2004). Gene expression profiling of gliomas strongly predicts survival. Cancer Res.

[122] Phillips HS, Kharbanda S, Chen R, Forrest WF, Soriano RH, Wu TD, Misra A, Nigro JM, Colman H, Soroceanu L, Williams PM, Modrusan Z, Feuerstein BG, Aldape K (2006). Molecular subclasses of high-grade glioma predict prognosis, delineate a pattern of disease progression, and resemble stages in neurogenesis. Cancer Cell.

[123] Lee Y, Scheck AC, Cloughesy TF, Lai A, Dong J, Farooqi HK, Liau LM, Horvath S, Mischel PS, Nelson SF (2008). Gene expression analysis of glioblastomas identifies the major molecular basis for the prognostic benefit of younger age. BMC Med. Genomics.

[124] (2008). Comprehensive genomic characterization defines human glioblastoma genes and core pathways. Nature.

[125] Li Y, Bollag G, Clark R, Stevens J, Conroy L, Fults D, Ward K, Friedman E, Samowitz W, Robertson M (1992). Somatic mutations in the neurofibromatosis 1 gene in human tumors. Cell.

[126] Mizoguchi M, Nutt CL, Mohapatra G, Louis DN (2004). Genetic alterations of phosphoinositide 3-kinase subunit genes in human glioblastomas. Brain Pathol.

[127] Cahill DP, Levine KK, Betensky RA, Codd PJ, Romany CA, Reavie LB, Batchelor TT, Futreal PA, Stratton MR, Curry WT, Iafrate AJ, Louis DN (2007). Loss of the mismatch repair protein MSH6 in human glioblastomas is associated with tumor progression during temozolomide treatment. Clin. Cancer Res.

[128] Hunter C, Smith R, Cahill DP, Stephens P, Stevens C, Teague J, Greenman C, Edkins S, Bignell G, Davies H, O’Meara S, Parker A, Avis T, Barthorpe S, Brackenbury L, Buck G, Butler A, Clements J, Cole J, Dicks E, Forbes S, Gorton M, Gray K, Halliday K, Harrison R, Hills K, Hinton J, Jenkinson A, Jones D, Kosmidou V, Laman R, Lugg R, Menzies A, Perry J, Petty R, Raine K, Richardson D, Shepherd R, Small A, Solomon H, Tofts C, Varian J, West S, Widaa S, Yates A, Easton DF, Riggins G, Roy JE, Levine KK, Mueller W, Batchelor TT, Louis DN, Stratton MR, Futreal PA, Wooster R (2006). A hypermutation phenotype and somatic MSH6 mutations in recurrent human malignant gliomas after alkylator chemotherapy. Cancer Res.

[129] Murat A, Migliavacca E, Gorlia T, Lambiv WL, Shay T, Hamou MF, de Tribolet N, Regli L, Wick W, Kouwenhoven MC, Hainfellner JA, Heppner FL, Dietrich PY, Zimmer Y, Cairncross JG, Janzer RC, Domany E, Delorenzi M, Stupp R, Hegi ME (2008). Stem cell-related “self-renewal” signature and high epidermal growth factor receptor expression associated with resistance to concomitant chemoradiotherapy in glioblastoma. J. Clin. Oncol.

